# Prediction of Cell-Penetrating Potential of Modified Peptides Containing Natural and Chemically Modified Residues

**DOI:** 10.3389/fmicb.2018.00725

**Published:** 2018-04-12

**Authors:** Vinod Kumar, Piyush Agrawal, Rajesh Kumar, Sherry Bhalla, Salman Sadullah Usmani, Grish C. Varshney, Gajendra P. S. Raghava

**Affiliations:** ^1^Center for Computational Biology, Indraprastha Institute of Information Technology, Okhla, India; ^2^Bioinformatics Centre, CSIR-Institute of Microbial Technology, Sector-39A, Chandigarh, India; ^3^Cell Biology and Immunology, CSIR-Institute of Microbial Technology, Sector-39A, Chandigarh, India

**Keywords:** modified cell-penetrating peptides, machine learning, Random Forest, SVM, *in silico* method, chemical descriptors, antimicrobial peptide

## Abstract

Designing drug delivery vehicles using cell-penetrating peptides is a hot area of research in the field of medicine. In the past, number of *in silico* methods have been developed for predicting cell-penetrating property of peptides containing natural residues. In this study, first time attempt has been made to predict cell-penetrating property of peptides containing natural and modified residues. The dataset used to develop prediction models, include structure and sequence of 732 chemically modified cell-penetrating peptides and an equal number of non-cell penetrating peptides. We analyzed the structure of both class of peptides and observed that positive charge groups, atoms, and residues are preferred in cell-penetrating peptides. In this study, models were developed to predict cell-penetrating peptides from its tertiary structure using a wide range of descriptors (2D, 3D descriptors, and fingerprints). Random Forest model developed by using PaDEL descriptors (combination of 2D, 3D, and fingerprints) achieved maximum accuracy of 95.10%, MCC of 0.90 and AUROC of 0.99 on the main dataset. The performance of model was also evaluated on validation/independent dataset which achieved AUROC of 0.98. In order to assist the scientific community, we have developed a web server “CellPPDMod” for predicting the cell-penetrating property of modified peptides (http://webs.iiitd.edu.in/raghava/cellppdmod/).

## Introduction

Since the existence of human race, therapeutic molecules have been used to cure human illness and to extend lives (Tosato et al., 2007). In past, thousands of molecules have been studied to combat deadly diseases. The ideal molecule must attain the desired therapeutic effect without causing side effects. A large number of promising therapeutic molecules disparage before reaching to its target (Gupta and Jhawat, 2017). In order to overcome this, several delivery vehicles have been discovered in last three decades, such as nanoparticle (Wang et al., 2018) and lipid carrier conjugate (Xu et al., 2017). Cell-penetrating peptide (CPP) is one of the most emergent and widely accepted drug delivery vehicle, having ability to internalize even into eukaryotic cells in non-disruptive way. These are short peptides of 3 to approximately 40 amino acids, mostly cationic followed by amphipathic in nature (Agrawal et al., 2016). CPPs can transport various biologically active molecules inside microbes as well as mammalian cells (Gao et al., 2014; Kurrikoff et al., 2016). CPPs such as TP10 and pVEC had been shown to significantly inhibit growth of few microbes as *Candida albicans, Staphylococcus aureus* as well as *Mycobacterium smegmatis* (Nekhotiaeva et al., 2004). CPPs and cationic antibacterial peptides have similar physicochemical properties, so many CPPs have shown antimicrobial activity (Splith and Neundorf, 2011; Bahnsen et al., 2013; Rodriguez Plaza et al., 2014). The poor membrane permeability of drug molecule always remains a concern in drug designing. In the era of drug resistance, where pathogen membrane provides a significant barrier, intracellular delivery of antibiotics/drugs by the virtue of CPP, proved to be a vital step in combating drug resistance to some extent (Sparr et al., 2013). CPP based conjugates (Ganguly et al., 2008; Jain et al., 2015) and combination therapy has been explored against several resistant pathogens (Randhawa et al., 2016). They have been proved effective against intracellular pathogens too (Gomarasca et al., 2017).

A universal mechanism of CPP internalization is always proved to be an exploring question, as the involved pathways are not fully clarified yet. The difficulty arises due to differing size, physicochemical properties, as well as concentration of diverse CPP and CPP-conjugates (Guidotti et al., 2017). Several mechanisms have been shown by various CPPs to translocate in to the cell, as micelle formation (Derossi et al., 1996), pore formation (Matsuzaki et al., 1996), membrane thinning (Pouny et al., 1992), endocytosis (Ferreira and Boucrot, 2018) and micropinocytosis (Jones, 2007). Majority of CPP internalization occurs via endocytosis, but several evidences suggest that at a threshold concentration direct penetration does occur (Palm-Apergi et al., 2012). CPPs can be used for intracellular delivery of small molecule-based drug (Lindgren et al., 2006), oligonucleotide (Margus et al., 2012), peptide and protein (Morris et al., 2001) and trans-epithelial delivery of peptides (Tan et al., 2014).

Despite, numerous properties and potential applications of CPPs, still there use in real life is limited. The primary limitation associated with CPP is endosomal compartment entrapment which reduces the bioavailability of the drug several times. In literature, it has been shown that bioavailability of CPPs can be increased several times by introducing a chemical modification in a CPP (Postlethwaite et al., 1996; Kim et al., 2006; Lundberg et al., 2007; Koppelhus et al., 2008; Aubry et al., 2009). N-terminal stearylation of Arg8 peptide improved the delivery of siRNA (Futaki et al., 2001), C-terminal cysteamidation of MPG peptide improved the delivery of siRNA (Simeoni et al., 2003), cysteine residue modification improved the stability of Tat peptide and thus enhances the plasmid delivery (Lo and Wang, 2008), Poly-L-ornithine modification in PepFect 14 peptide increases transfection efficiency of oligonucleotide in HeLa pLuc 705 (Ezzat et al., 2011). Thus, it is important to understand chemical modification of residues in a peptide and its impact on cell-penetrating property of peptides.

In the last few years, several computational methods have been developed for the prediction of CPPs. These methods have been developed on various features like amino acid composition (Sanders et al., 2011), dipeptide composition (Tang et al., 2016), binary profile, physiochemical properties and motifs (Gautam et al., 2013). They have also applied Z-scale based method (Sandberg et al., 1998), feature selection techniques (Tang et al., 2016), classifiers like Random Forest (RF) (Wei et al., 2017), Support Vector Machine (SVM) (Sanders et al., 2011). Beside this, few more methods have been developed in recent years for predicting CPPs with high accuracy (Chen et al., 2015; Tang et al., 2016; Wei et al., 2017). Best of authors knowledge, all methods developed so far for predicting CPPs are suitable for peptides containing natural residues only, but no method has been developed for predicting cell penetration property of peptides with non-natural and modified residues. In this study, a systematic attempt has been made to develop a machine learning method for predicting cell penetration ability of peptides containing non-natural and modified residues. Machine learning technique derive features/rules from the experimentally validated modified CPPs and Non-CPPs are used to predict cell penetration ability of a modified peptide. We hope this method will be useful for researchers working in the field of drug delivery.

## Materials and methods

### Creation of dataset for CPPs and non-CPPs

Cell-penetrating peptides were extracted from CPPsite2.0 database (Agrawal et al., 2016), which provides comprehensive information on wide-range of CPPs. It consists of 1,850 experimentally validated natural and modified CPPs. We remove CPPs that does not contain any modified residue; we also remove peptides whose tertiary structure is not available in the database. Finally, we got 732 chemically modified CPPs whose structure is available in CPPsite 2.0. We assign this set of 732 CPPs as positive set or set of CPPs. To develop any method, we also need equal number of negative examples. In this study, we extracted non-CPPs from SATPdb (Singh et al., 2016) database which maintains information of 19,192 peptides having several properties. We extracted structures of 732 peptides, which may exhibit any characteristic other than cell penetrating property. This set of peptides were assigned as negative set or set of non-CPPs. Finally, we built the dataset that contains 732 CPPs and 732 non-CPPs whose sequence and tertiary structure is available in CPPsites 2.0 or SATPdb.

### Datasets for internal and external validation

The dataset was divided into two datasets namely training (main) and validation dataset (Bhalla et al., 2017). The training dataset consists of 80% of peptides, 582 CPPs, and 582 non-CPPs. The validation dataset consists of remaining 20% of peptides, 150 CPPs, and 150 non-CPPs. We used training dataset for developing models and for internal validation. In internal validation, models were trained and tested using commonly used five-fold cross-validation technique (Nagpal et al., 2017). Performance of best model achieved on training dataset, was evaluated on validation dataset. The evaluation of the performance of model on validation or independent dataset is called external validation.

### Model development

#### Computation of features from peptide structures

##### Composition based features

Atom composition is computed from CPPs and non-CPPs by converting peptide structures in SMILES format using openbabel (O'Boyle et al., 2011). These SMILES were further used to compute atom composition of following atoms C, H, O, N, S, Cl, Br, and F. The atomic composition provided the fixed length of 8 vectors.

$$\begin{array}{r} Fraction\;of\;atom\left(a\right)=\frac{Total\;number\;of\;atom\left(a\right)}{Total\;number\;of\;all\;possible\;diatoms}\times 100 \end{array}$$

Where atom (*a*) is one out of 8 atoms.

##### Diatom composition

We computed diatom composition of amino acids just like the atomic composition for CPPs and non-CPPs. The diatomic composition provides the composition of the pair of atoms in each residue (e.g., C-C, C-O, etc.) of the peptide, and used to convert the variable length of modified peptides to fixed length feature vectors. The diatomic composition provided the fixed length of 64 (8 × 8) vectors.

$$\begin{array}{r} Fraction\;of\;Diatom\left(a\right)=\frac{Total\;number\;of\;Diatom\left(a\right)}{Total\;number\;of\;all\;possible\;diatoms}\times 100 \end{array}$$

Where diatom (*a*) is one out of 64 diatoms.

##### Chemical descriptors

A biological property of any chemical molecule is determined by its chemical descriptors, which have been used in the past to develop QSAR based molecules (Kumar et al., 2015). PaDEL software, a freely available software was used for the calculation of chemical descriptors (Yap, 2011). We calculated 15,537 different types of descriptors, including 2D, 3D, and 10 different types of fingerprints. As all descriptors don't correlate with biological activity, we have done feature selection using “CsfSubsetEval” function present in WEKA software (Smith and Frank, 2016) to remove unnecessary descriptors hence reduced noise from dataset.

### Computation of features from amino acid sequence of peptide

#### Amino acid composition

We substitute the symbol of the modified residue with its original natural amino acid, for calculating amino acid composition for the positive and negative dataset. This left us with the sequence having 20 natural amino acids which generated the vector of 20.

$$\begin{array}{r} AAC\left(a\right)=\frac{Ra}{N}x100 \end{array}$$

Here, *AAC* (*a*) is the percent composition of amino acid (a); *R*_*a*_ is the numbers of residues of type a, and *N* represents the total number of peptide's residues.

#### Dipeptide composition

We also calculated dipeptide composition of the peptides since it provides global information of the peptide. The dipeptide composition was calculated using the formula 4, and it generated the vector of 400 (20 × 20).

$$\begin{array}{r} Fraction\;of\;Dipeptide\;\left(a\right)=\frac{Total\;number\;of\;Dipeptide\left(a\right)}{Total\;number\;of\;all\;possible\;dipeptides}\times 100 \end{array}$$

Where dipeptide (*a*) is one out of 400 dipeptides.

#### Terminus composition-based model

We also calculated N and C terminus amino acid composition as well as dipeptide composition for developing prediction models. The composition of 5, 10, and 15 residues from N-terminus as well as C-terminus was taken into account. Also, we joined the terminal residues like N5C5, N10C10, and N15C15 and for developing models.

### Residue preference

In order to observe the residue preference at a particular position in the peptide, web-logos were prepared for first 15 N and 15 C-terminals along with their modifications using online WebLogo software (Crooks et al., 2004). These logos provide the position specific frequency of amino acids in a peptide. Each logo consists of stacks of symbols, one stack for each position in the sequence. The overall height of the stack indicates the sequence conservation at that position while the height of symbols within the stack indicates the relative frequency of each amino acid at that position.

### Statistical analysis

To check whether is there any significance difference between modified CPPs and non-CPPs, we performed Welch *t*-test on the selected features of 2D, 3D and Fingerprints descriptors using in house R-script. Adjusted *p*-values were calculated using Boneferroni adjustment.

### Performance measure

Different parameters were used to check the performance of various models developed in this study. These parameters are divided into two groups.

#### Threshold dependent parameters

This category includes Sensitivity (Sen), Specificity (Spc), Accuracy (Acc), and Matthews's correlation coefficient (MCC), where Sensitivity is true positive rate, Specificity is true negative rate, accuracy is ability to differentiate true positive and true negative and MCC is a correlation coefficient between observed and predicted. These can be calculated using the following equations.

$$\begin{array}{r} \text{Sensitivity}=\frac{TP}{PS}\times 100 \end{array}$$

$$\begin{array}{r} \text{Specificity}=\frac{TN}{NS}\times 100 \end{array}$$

$$\begin{array}{r} \text{Accuracy}=\frac{TP+TN}{PS+NS}\times 100 \end{array}$$

$$\begin{array}{r} \text{MCC}=\frac{1-\left(\frac{FN}{PS}\times \frac{FP}{NS}\right)}{\sqrt{\left(1+\frac{FP-FN}{PS}\right)\times \left(1+\frac{FN-FP}{NS}\right)}} \end{array}$$

Where *TP* represents correctly predicted positive, *TN* represents the correctly predicted negative examples, *PS* represents total sequences in positive set, *NS* represents total sequences in negative set, *FP* represents actual negative examples which have been wrongly predicted as positive and *FN* represents wrongly predicted positive examples. This is a well-established method of measuring performance and has been used earlier in many studies (Porto et al., 2017; Agrawal et al., 2018).

#### Threshold independent parameters

In this study, we also used threshold independent measure to evaluate the performance of models. In case of threshold independent measures, Receiver Operating Characteristics (ROC) curve is drawn between false positive and false negative rates. In order to measure performance, Area Under Curve ROC curve is computed called AUROC.

## Results

### Analysis

**Figure 1:**
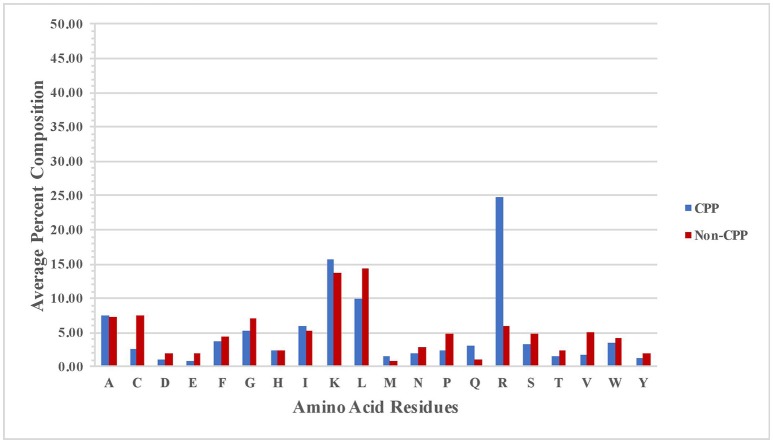
Percentage amino acid composition of CPPs and non-CPPs.

We compute percent average composition of atoms in CPPs and non-CPPs to understand the preference of certain types of atoms present in the CPPs and non-CPPs. Overall, the profile is more or less same in both CPPs and non-CPPs.CPPs are slightly rich in H and N atoms whereas non-CPPs are slightly rich in C, O, and S (Figure S1). We analyzed the amino acid composition of both positive (CPPs) and negative (Non-CPPs) dataset. It has been observed that certain type of residues like R, K, and Q are more prominent in CPPs; in contrast residues are like C, L, V, P, and G are not preferred in CPPs (Figure 1). In the same manner, we also calculated the average amino acid composition of the first 15 N and 15 C- terminal amino acid residues (Figure S2). At the N terminal R, Q, I and M are more prominent in CPP as compared to Non-CPP (Figure S2A). Similarly at C terminal, R, K and Q are more prominent (Figure S2B).

**Figure 2:**
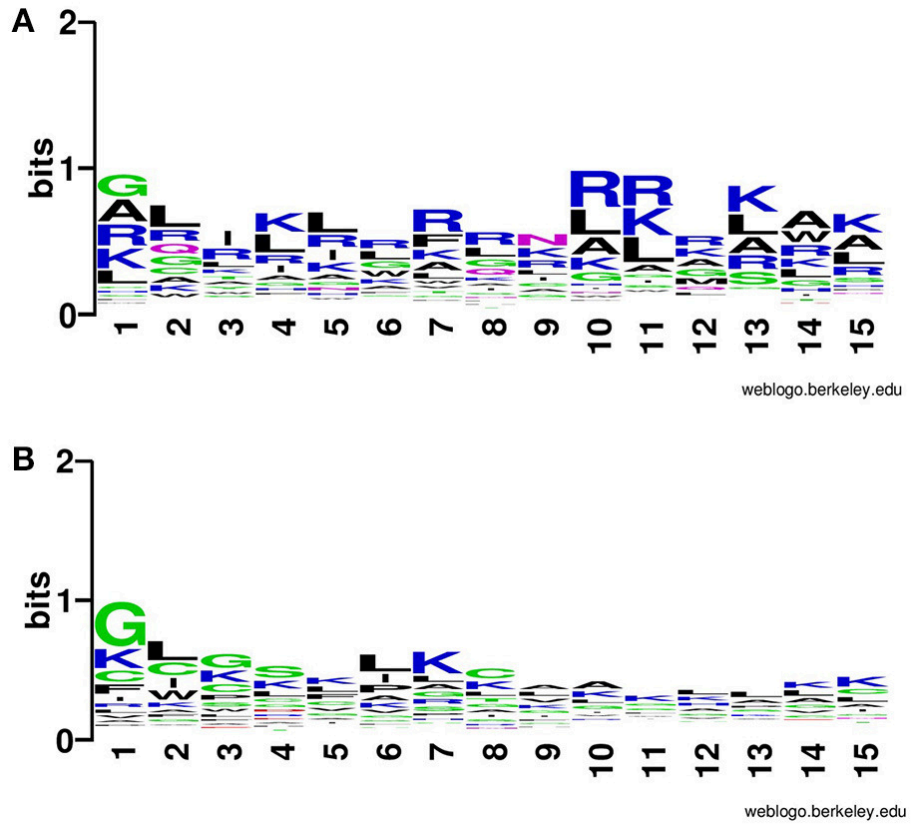
Weblogo illustrating residue preference of first 15 N terminal residues of modified **(A)** CPPs and **(B)** non-CPPs.

**Figure 3:**
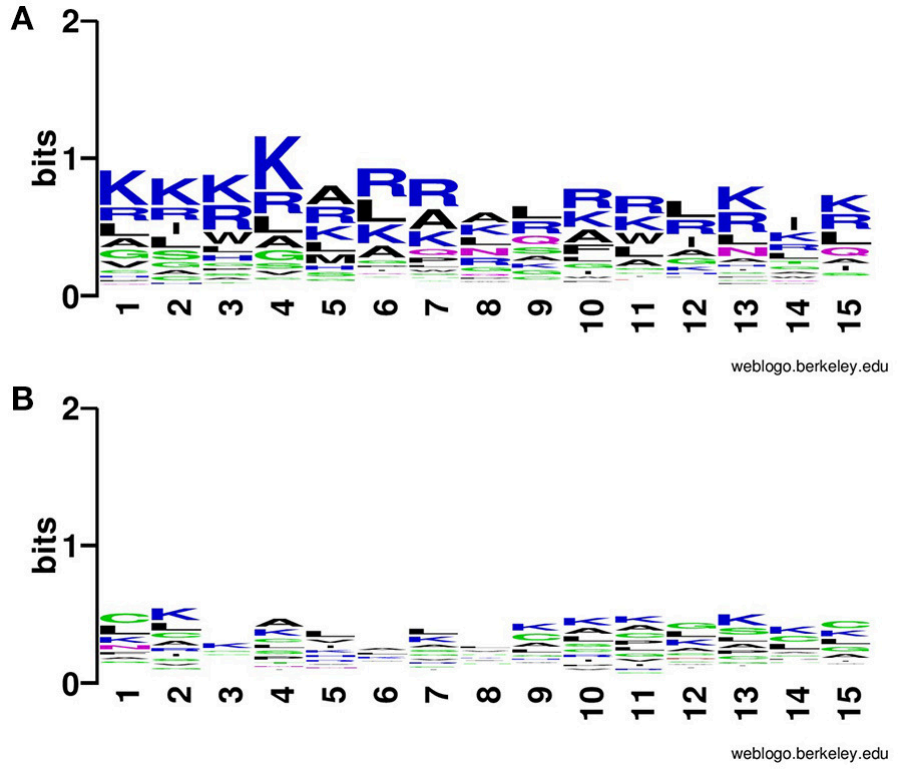
Weblogo illustrating residue preference of first 15 C terminal residues of modified **(A)** CPPs and **(B)** non-CPPs.

In addition to compositional preference, we also computed preference of different types of residues in CPPs. It was revealed that some specific type of residues was preferred in the positive dataset contain CPPs as compared to the negative dataset contain non-CPPs. Residues like Rand K are highly preferred at various positions CPPs particularly at N terminal (Figure 2). Similarly, K and R are mostly preferred at C terminal also (Figure 3).

### Machine learning based prediction model

We used various machine-learning approaches like SVM, Random Forest, Naive Bayes, J48 and SMO for developing the prediction model. These models utilize different features or descriptors to discriminate or classify CPPs and non-CPPs. The results are explained in details in the following sections.

#### Model based on peptide structure

Tertiary structure of a peptide can present all type of modifications. Thus structure of peptide is used to predict cell penetration ability of modified peptide. In this study, we got structure of peptides from databases CPPsite 2.0 and SATPdb. The models were developed using various features of peptide structures. First, we developed model using atomic composition of peptides. In order to obtain atomic composition of peptides from its structure, we convert structure from sdf format to SMILES. The atomic composition of peptides was calculated from SMILES of peptide. Prediction models were developed using different classifiers like SVM, RF, Naive Bayes, SMO and J48 using atomic composition as an input feature. Random Forest based classification model provided the highest accuracy of 84.02%, MCC of 0.68 and AUROC of 0.91 on the training dataset. On validation dataset, we achieved maximum accuracy of 78.33%, MCC of 0.57 and AUROC of 0.88. Performance of different classifiers given in Table 1. We also developed model using diatom composition of peptides and obtained the highest accuracy of 88.40% with MCC of 0.77. On validation dataset, we achieved maximum accuracy of 91.00% with MCC 0.83. Here SVM based model performed best among all the classifiers used for prediction (Table 2).

**Table 1:** Performance of different machine learning methods on atom composition.

	**Parameter**	**Main dataset**					**Validation dataset**				
		**Sen**	**Spc**	**Acc**	**MCC**	**AUROC**	**Sen**	**Spc**	**Acc**	**MCC**	**AUROC**
SVM	*g* = 1, *c* = 9, j = 4	81.10	80.58	80.84	0.62	0.84	79.33	75.33	77.33	0.55	0.81
Random Forest	Ntree = 30	83.33	84.71	84.02	0.68	0.91	79.33	77.33	78.33	0.57	0.88
SMO	*g* = 1, *c* = 2	77.66	83.51	80.58	0.61	0.80	75.33	82.67	79.00	0.58	0.79
J48	*c* = 0.1, *m* = 1	75.43	80.58	78.01	0.56	0.82	80.00	76.00	78.00	0.56	0.79
Naive Bayes	Default	74.57	65.46	70.02	0.40	0.80	80.00	69.33	74.67	0.50	0.82

**Table 2:** Performance of different machine learning methods on diatom composition.

	**Parameters**	**Main dataset**					**Validation dataset**				
		**Sen**	**Spc**	**Acc**	**MCC**	**AUROC**	**Sen**	**Spc**	**Acc**	**MCC**	**AUROC**
SVM	*g* = 0.01, *c* = 15, j = 2	90.38	86.43	88.40	0.77	0.93	85.33	96.67	91.00	0.83	0.97
Random Forest	Ntree = 30	88.49	88.49	88.49	0.77	0.94	85.33	82.00	83.67	0.67	0.93
SMO	*g* = 0.1, *c* = 4	86.25	89.00	87.63	0.75	0.87	86.67	84.00	85.33	0.71	0.85
J48	*c* = 0.3, *m* = 1	82.47	81.10	81.79	0.64	0.81	85.33	81.33	83.33	0.67	0.82
Naive Bayes	Default	71.65	70.45	71.05	0.42	0.78	72.67	66.67	69.67	0.39	0.77

We developed models individually for 2D descriptors, 3D descriptors, and Fingerprints as well as the single model by combining 2D, 3D descriptors, and Fingerprints. The descriptors were computed using PaDEL software from tertiary structure of peptides (sdf format). The models were developed on the features, selected after performing feature selection, by attribute evaluator named, “CfsSubsetEval” with search method of “BestFirst” at default parameters in the forward direction (amount of backtracking, *N* = 5 and lookup size *D* = 1). In case of 2D descriptors, total 144 descriptors were calculated initially and were reduced to 17 after feature selection. List of the selected features is provided in Table S1. We applied different machine learning techniques on these selected features and observed that Random Forest based model achieved the maximum accuracy of 92.34%, MCC of 0.85 and AUROC of 0.97 for the main dataset and 91.67% accuracy, 0.83 MCC and 0.97 AUROC for the validation dataset (Table 3).

**Table 3:** Performance of different machine learning methods on 2D descriptors.

	**Parameters**	**Main dataset**					**Validation dataset**				
		**Sen**	**Spc**	**Acc**	**MCC**	**AUROC**	**Sen**	**Spc**	**Acc**	**MCC**	**AUROC**
SVM	*g* = 0.01, *c* = 6, j = 2	89.00	84.48	86.75	0.74	0.92	86.00	82.67	84.33	0.69	0.92
Random Forest	Ntree = 60	92.78	91.90	92.34	0.85	0.97	94.67	88.67	91.67	0.83	0.97
SMO	*g* = 0.001, *c* = 4	83.16	86.38	84.77	0.70	0.84	81.33	87.33	84.33	0.69	0.84
J48	*c* = 0.2, *m* = 2	89.52	88.79	89.16	0.78	0.89	90.00	87.33	88.67	0.77	0.89
Naive Bayes	Default	75.09	78.79	76.94	0.54	0.85	74.67	77.33	76.00	0.52	0.84

In case of 3D descriptors, total 47 features were calculated and was reduced to 6 after applying feature selection (Table S2). On these features, Random Forest model performed better than other models and achieved maximum accuracy of 76.55%, MCC of 0.53 and AUROC value of 0.85 on the main dataset and 73.49% accuracy, 0.47 MCC and 0.83 AUROC on validation dataset (Table 4). The different types of fingerprints generated 14,532 features, which were reduced to 27 after feature selection (Table S3). Performance of different classifiers were evaluated on these features (Table 5) and once again Random Forest showed the best performance with maximum accuracy of 92.25%, MCC of 0.85 and AUROC of 0.98 on main dataset and accuracy of 92.33%, MCC of 0.85 and AUROC of 0.98 on validation dataset.

**Table 4:** Performance of different machine learning methods on 3D descriptors.

	**Parameters**	**Main dataset**					**Validation dataset**				
		**Sen**	**Spc**	**Acc**	**MCC**	**AUROC**	**Sen**	**Spc**	**Acc**	**MCC**	**AUROC**
SVM	*g* = 1e-05, *c* = 15, *j* = 1	76.29	74.40	75.34	0.51	0.80	71.14	73.15	72.15	0.44	0.80
Random Forest	Ntree = 700	80.93	72.16	76.55	0.53	0.85	79.87	67.11	73.49	0.47	0.83
SMO	*g* = 0.0005, *c* = 2	69.42	72.85	71.13	0.42	0.71	63.09	76.51	69.80	040	0.69
J48	*c* = 0.1, *m* = 3	74.74	76.12	75.43	0.51	0.78	72.48	74.50	73.49	0.47	0.78
Naive Bayes	Default	69.24	74.40	71.82	0.44	0.78	69.80	75.84	72.82	0.46	0.79

**Table 5:** Performance of different machine learning methods on fingerprints.

	**Parameters**	**Main dataset**					**Validation dataset**				
		**Sen**	**Spc**	**Acc**	**MCC**	**AUROC**	**Sen**	**Spc**	**Acc**	**MCC**	**AUROC**
SVM	*g* = 0.005, *c* = 15, *j* = 1	90.19	88.12	89.16	0.78	0.95	93.33	89.33	91.33	0.83	0.96
Random Forest	Ntree = 600	94.32	90.19	92.25	0.85	0.98	96.67	88.00	92.33	0.85	0.98
SMO	*g* = 0.0005, *c* = 4	85.54	85.03	85.28	0.71	0.85	88.67	85.33	87.00	0.74	0.87
J48	*c* = 0.25, *m* = 1	90.02	89.33	89.67	0.79	0.89	88.67	88.67	88.67	0.77	0.90
Naive Bayes	Default	86.40	84.34	85.37	0.71	0.90	82.67	85.33	84.00	0.68	0.90

**Figure 4:**
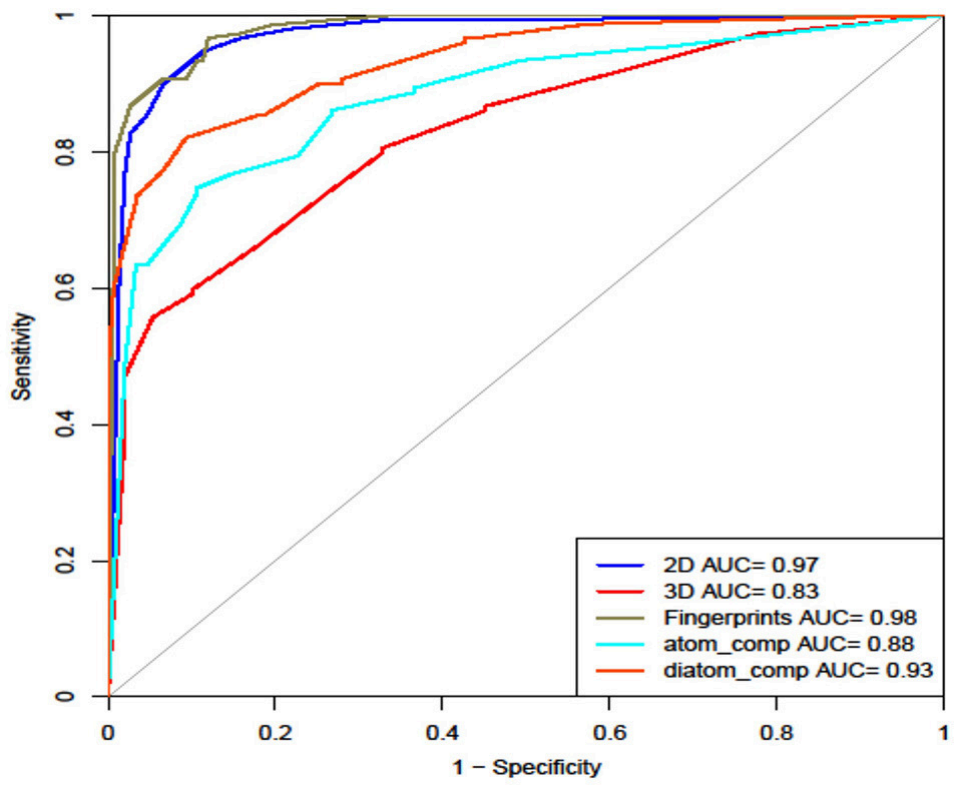
ROC curve showing performance of models on various structural features.

Finally, we calculated all the 2D, 3D descriptors and fingerprints at the same time, which generated 15,204 features. Feature selection reduced it down to 48 important features on which different machine learning classifiers were evaluated. Here we observe the maximum accuracy of 95.10%, MCC of 0.90 and AUROC of 0.99 on main dataset and 92.33% accuracy, 0.85 MCC and 0.98 AUROC on validation dataset by Random Forest model (Table 6). Figure 4 shows the AUROC curve as well as AUROC values of different models.

**Table 6:** Performance of different machine learning methods on 2D, 3D and fingerprints collectively.

	**Parameters**	**Main dataset**					**Validation dataset**				
		**Sen**	**Spc**	**Acc**	**MCC**	**AUROC**	**Sen**	**Spc**	**Acc**	**MCC**	**AUROC**
SVM	*g* = 1e-05, *c* = 15, *j* = 1	83.33	79.21	81.27	0.63	0.89	78.67	82.67	80.67	0.61	0.87
Random Forest	Ntree = 60	95.19	95.02	95.10	0.90	0.99	91.33	93.33	92.33	0.85	0.98
SMO	*g* = 0.0001, *c* = 5	76.80	76.98	76.89	0.54	0.76	75.33	83.33	79.33	0.59	0.79
J48	*c* = 0.25, *m* = 5	89.69	87.63	88.66	0.77	0.90	84.67	92.00	88.33	0.77	0.92
Naive Bayes	Default	95.19	88.14	91.67	0.84	0.95	92.00	89.33	90.67	0.81	0.96

##### Significance of features

We obtained significant difference between the positive and negative features based on adjusted *p*-values. *P*-values were found to be less than 0.05 for most of the features. Therefore, we can say that these features can be used to discriminate modified CPPs and non-CPPs. Mean value of positive and negative features along with their *p*-value for 2D, 3D, and fingerprint descriptors is provided in Tables S1–S3.

### Model based on peptide sequence

It is nearly impossible to present a modified peptide by amino acid sequence. Thus, prediction of modified peptide from there sequence is not possible. Same time generating tertiary structure of a peptide is a tedious job for a biologist. We made an attempt to develop prediction model for cell penetration peptides of modified peptides from their amino acid sequence only by ignoring modifications in peptide. First, we developed simple composition-based models using various machine learning techniques. The SVM based model showed the best performance among all the classifiers used in the study. The accuracy of 91.67%, MCC of 0.83 and AUROC of 0.96 was achieved for the main dataset. On validation dataset, we obtained accuracy of 89.67%, MCC of 0.79 and AUROC of 0.96 (Table S4). We also developed SVM based model on first 5, 10, and 15 N and C-terminus residues. Results are given in Table S5.

Secondly, we developed models using dipeptide composition, SVM classifier showed the highest accuracy of 91.84%, MCC of 0.84 and AUROC of 0.96 for the main dataset. For independent dataset, the accuracy of 92.33%, MCC of 0.85 and AUROC of 0.97 was achieved (Table S6). Results of SVM based models on terminus residues for dipeptide composition is provided in Table S7. It is important for users to understand that sequence based model is not alternate to structure based models or alternate to past sequence based models developed for natural peptides. This sequence based is just approximate cell penetration potential of a modified peptide from its amino acid sequence.

### Implementation of webserver

To assist the scientific community, the best models are provided freely at http://webs.iiitd.edu.in/raghava/cellppdmod/. The “PREDICTION” module, consider tertiary structure (PDB format) of the modified peptide as an input and does the prediction. If a user has no structural information, he/she can generate PDB structure of their peptide up to 25 residues in length using server “PEPstrMOD” (Singh et al., 2015) (http://webs.iiitd.edu.in/raghava/pepstrmod/) developed by our group specifically for predicting the structure of the modified peptide. In case of natural peptide user can also use following servers PEP-FOLD (Thevenet et al., 2012) (http://bioserv.rpbs.univ-paris-diderot.fr/services/PEP-FOLD/) and QUARK (Xu and Zhang, 2012) (https://zhanglab.ccmb.med.umich.edu/QUARK/) for predicting structure of peptides. Multiple modification options are provided there, and the user can choose the desired modification. After generating the structure, user can do the prediction on “PREDICTION” module, whether the given modified PDB structure is CPP or non-CPP. Beside the main model, we have also implemented model based on peptide sequence (Subsidiary model). We have also provided a “DOWNLOAD” module from where the user can download the dataset used in this study.

## Discussion

CPPs has shown a promising impact in the field of therapeutics or for targeting a specific disease (Bechara and Sagan, 2013). However, the major limitations associated with some of these CPPs is their entrapment of CPP-cargo in endosomal compartments followed by endocytosis and therefore their bioavailability and half-life is severely reduced (Mäe et al., 2009). To overcome this limitation, people have tried to modify the CPP chemically. For example, to increase the delivery of nucleic acid more efficiently, people have introduced chemical modifications like N terminal stearylation (Futaki et al., 2001; Khalil et al., 2004), C-terminal cysteamidation (Simeoni et al., 2003; Morris et al., 2007), residue modifications (Lundberg et al., 2007). Tat is one of the first CPP, discovered from protein of HIV and various studies showed that it enhances the uptake of various drug and protein (Brooks et al., 2005). But DNA delivery by Tat is limited, because of the instability of Tat-DNA complex (Lo and Wang, 2008). Lo and Wang (2008) showed that Cysteine makes the Tat-DNA complex more stable. Incorporation of two cysteine residues results into interpeptide disulphide bond, form by air oxidation once bind to DNA. This enhance the stability of Tat-DNA complex, as well as protect DNA in extracellular environment. Therefore, gene transfection efficiency is more in modified Tat than simple Tat.

Computational algorithms have been proved a wide success in designing therapeutic peptides (Dhanda et al., 2017), therefore a large number of sequence-based model to design CPP has been developed in past. But all of these models have one limitation in common that they can only handle peptides with natural residues. Due to the huge therapeutic importance of modified CPP, prediction and designing of modified CPPs is the need of hour. So, we have developed a computational method, which is based on structural features, can handle the natural as well as modified peptides both. Beside this we have also incorporated a subsidiary model based on the sequence of peptides which consider only natural residues, to handle large number of peptides simultaneously. Here, sequence-based model is not alternate to the methods developed in past to predict natural CPPs.

We have developed various models using machine learning techniques such as SVM, Random Forest, J48, naïve bayes, SMO; individually for atom composition, 2D descriptors, 3D descriptors, and Fingerprints as well as the single model by combining 2D, 3D descriptors, and Fingerprints. We obtain best performance by Random Forest for both combined (2D, 3D, and Fingerprint descriptors) as well as fingerprint with accuracy 92.33% and AUROC 0.98 on validation dataset. As fingerprint alone will be computationally more feasible as compared to the combined method, so we have implemented this model on webserver.

We believe this work will prove a great assist to the researchers aim to design cell penetrating peptide, as well as incorporate different modification and to check their effect on cell penetration ability. In future, we can improve this method, if better art of structure prediction will be developed, as right now PEPstrMOD could tackle only 7–25 amino acid length and other best model I-TASSER only deals with natural residues. So, in conclusion this field must grow simultaneously with the betterment of art-of-structure prediction.

## Author contributions

VK and PA generated the dataset. VK, PA, RK, and SB performed the experiments. VK, PA, and RK performed data analysis and prepared the tables and figures. VK, PA, RK, SB, and SU developed the web interface. VK, RK, PA, SU, and GR write the manuscript. GR and GV conceived the idea and coordinated the project.

### Conflict of interest statement

The authors declare that the research was conducted in the absence of any commercial or financial relationships that could be construed as a potential conflict of interest.

## Supplementary material

Figure S1 Percentage atomic composition of modified CPPs and non-CPPs.

Figure S2 Percentage amino acid composition of CPPs and non-CPPs **(A)** 15 N-terminal residues and **(B)** 15 C-terminal residues.

Table S1 List of 2D features with their positive mean value, negative mean value and *p*-value.

Table S2 List of 3D features with their positive mean value, negative mean value and *p*-value.

Table S3 List of fingerprints with their positive mean value, negative mean value and *p*-value.

Table S4 Performance of different machine learning methods on amino acid composition.

Table S5 Performance of SVM method on amino acid composition features of terminus residues.

Table S6 Performance of different machine learning methods on dipeptide composition.

Table S7 Performance of SVM method on dipeptide composition features of terminus residues.
