# Supplementary figures and images for: Prediction of Cell-Penetrating Potential of Modified Peptides Containing Natural and Chemically Modified Residues
Source: Front Microbiol. 2018 Apr 12;9:725. doi: 10.3389/fmicb.2018.00725 (PMC5906597; doi:10.3389/fmicb.2018.00725)

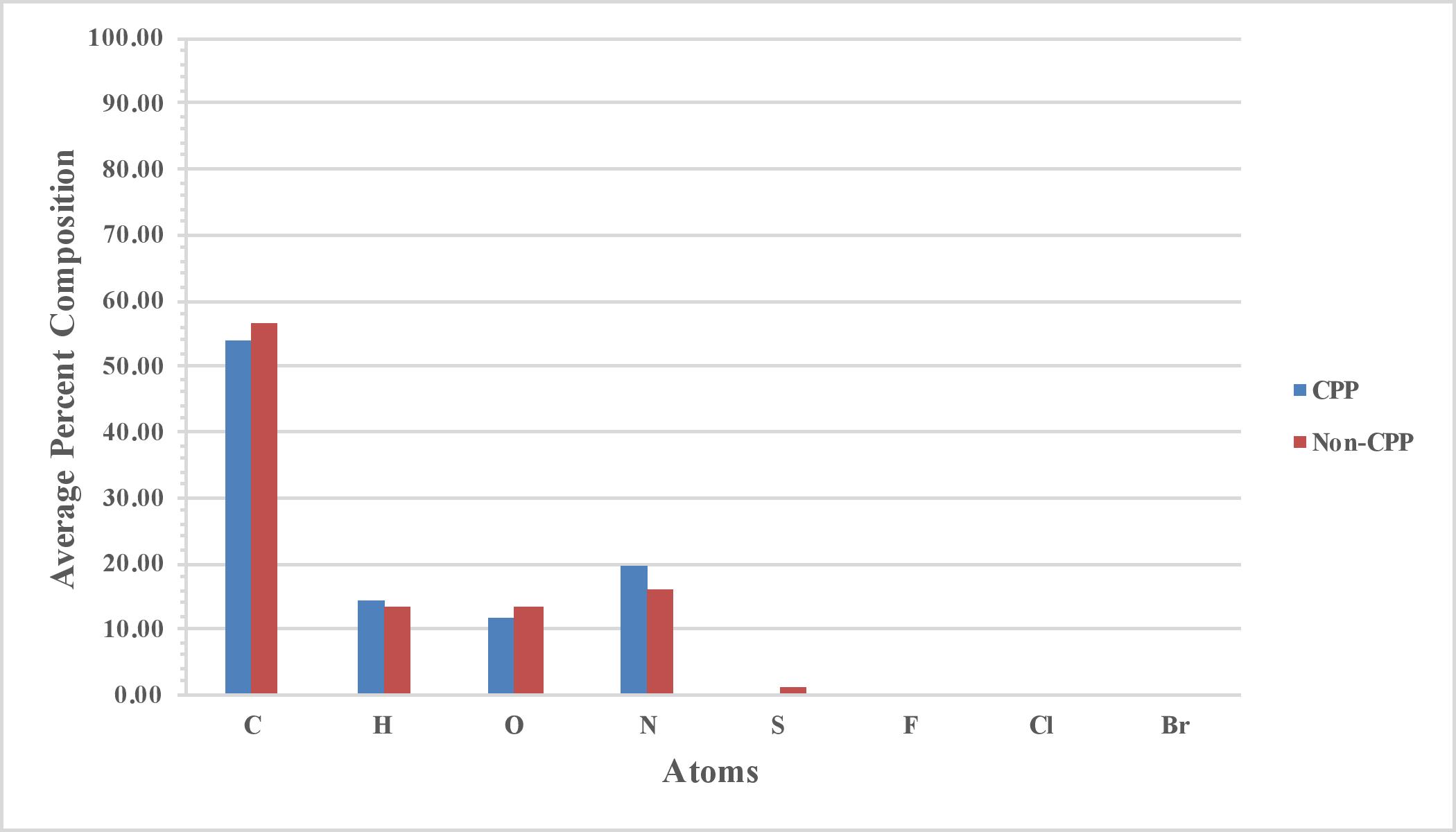

Supplement: Figure S1 — Percentage atomic composition of modified CPPs and non-CPPs. [file Image1.JPEG]

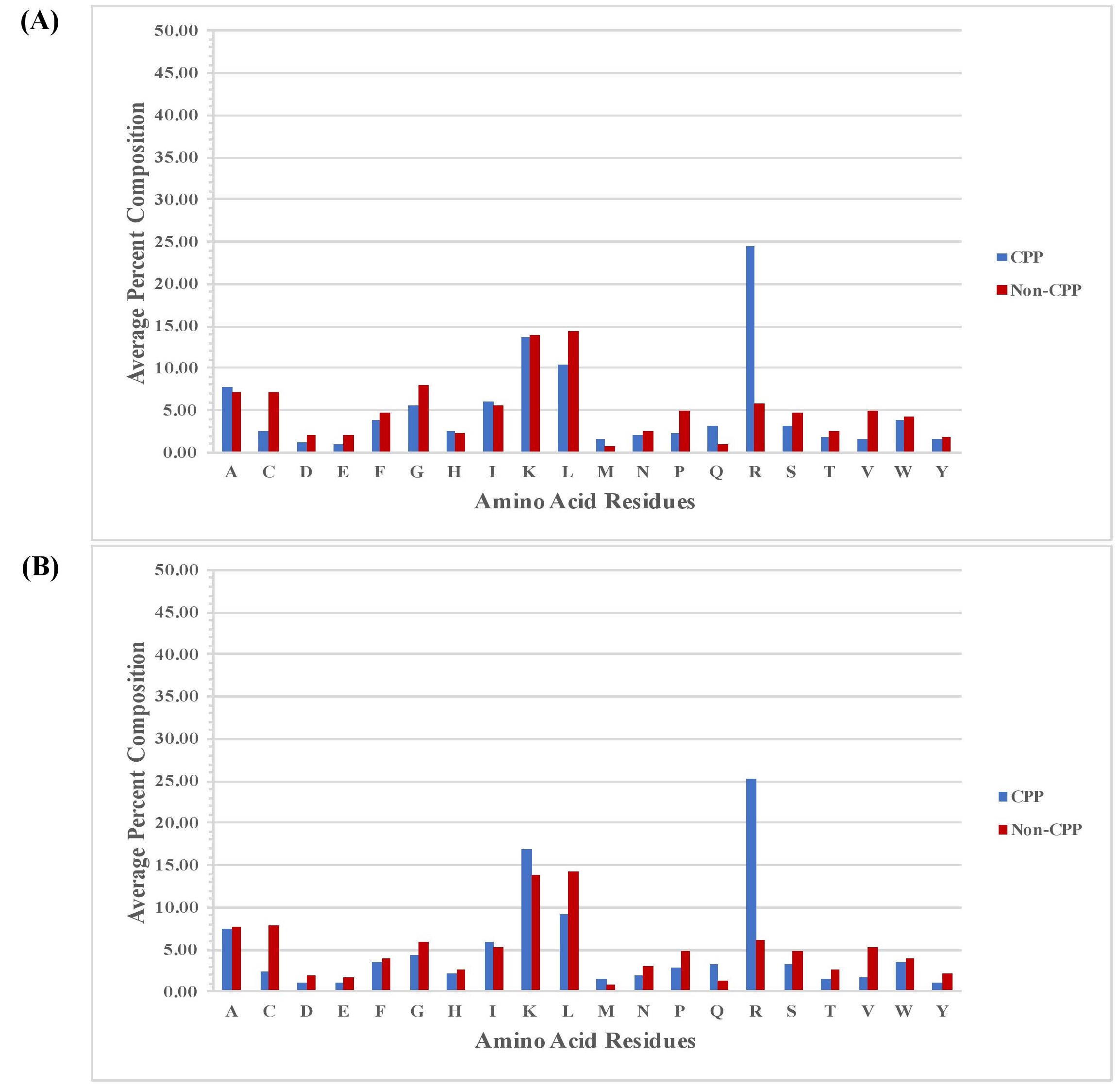

Supplement: Figure S2 — Percentage amino acid composition of CPPs and non-CPPs (A) 15 N-terminal residues and (B) 15 C-terminal residues. [file Image2.JPEG]
