# Supplementary material for: Prediction of Cell-Penetrating Potential of Modified Peptides Containing Natural and Chemically Modified Residues
Source: Front Microbiol. 2018 Apr 12;9:725. doi: 10.3389/fmicb.2018.00725 (PMC5906597; doi:10.3389/fmicb.2018.00725)
Supplement: Table S1 — List of 2D features with their positive mean value, negative mean value and p-value. [file DataSheet1.DOC]

**Prediction of cell-penetrating potential of modified peptides containing natural and chemically modified residues**

**Vinod Kumar^#,1,2^, Piyush Agrawal^#,1,2^, Rajesh Kumar^#,1,2^, Sherry Bhalla^1^, Salman Sadullah Usmani^1,2^, Grish C. Varshney^2^ and Gajendra P.S. Raghava^*,1,2^**

1. Center for Computational Biology, Indraprastha Institute of Information Technology, Okhla, New Delhi-110020, India.
2. Bioinformatics Centre, CSIR-Institute of Microbial Technology, Sector-39A, Chandigarh- 160036, India.

# Equal contribution

**^*^ Corresponding author**

Professor, Center for Computational Biology, Indraprastha Institute of Information Technology, Okhla, New Delhi 110020, India.

India. Tel.: +91 011 26907444, Fax No. +91 11 26907405

**E-mail address:** [**raghava@iiitd.ac.in**](mailto:raghava@iiitd.ac.in)

**Supplementary Information**

**Table S1. List of 2D features with their positive mean value, negative mean value and p-value.**

| **Sr. No.** | **Feature** | **Average_Mean_Pos** | **Average_Mean_Neg** | **P-value** |
| --- | --- | --- | --- | --- |
| 1 | nR | 3.371232877 | 0.797260274 | 0.000 |
| 2 | nC | 0.434246575 | 1.38630137 | 0.000 |
| 3 | nL | 3.868493151 | 4.512328767 | 0.179 |
| 4 | ATSc3 | -0.180160945 | -0.348503182 | 0.000 |
| 5 | khs.sCH3 | 8.356164384 | 10.18082192 | 0.000 |
| 6 | khs.dCH2 | 0.028767123 | 0 | 0.236 |
| 7 | khs.ssCH2 | 31.56164384 | 24.91369863 | 0.000 |
| 8 | khs.dsCH | 0.316438356 | 0.080821918 | 0.000 |
| 9 | khs.aaCH | 7.173972603 | 8.015068493 | 0.356 |
| 10 | khs.sNH2 | 5.72739726 | 3.068493151 | 0.000 |
| 11 | khs.sSH | 0.4 | 1.197260274 | 0.000 |
| 12 | nAtomLC | 119.5520548 | 89.52328767 | 0.000 |
| 13 | MDEN-11 | 1.540135295 | 0.787151028 | 0.000 |
| 14 | MDEN-12 | 4.148320521 | 4.261348991 | 1.000 |
| 15 | MDEN-13 | 0.224564364 | 0.200538681 | 1.000 |
| 16 | nG12Ring | 0.024657534 | 0.219178082 | 0.000 |
| 17 | nTG12Ring | 0.109589041 | 0.883561644 | 0.000 |

**Table S2. List of 3D features with their positive mean value, negative mean value and p-value.**

| **Sr. No.** | **Feature** | **Average_Mean_Pos** | **Average_Mean_Neg** | **P-value** |
| --- | --- | --- | --- | --- |
| 1 | PPSA-3 | 118.2476367 | 82.31044938 | 0.000 |
| 2 | DPSA-1 | 1231.080368 | 971.1035673 | 0.000 |
| 3 | FPSA-1 | 0.770632621 | 0.743481702 | 0.000 |
| 4 | FPSA-3 | 0.05130554 | 0.041553475 | 0.000 |
| 5 | WPSA-3 | 294.1959924 | 178.2647114 | 0.000 |
| 6 | RPCS | 0.054064437 | 0.042254736 | 6.553 |

**Table S3. List of fingerprints with their positive mean value, negative mean value and p-value.**

| **Sr. No.** | **Feature** | **Average_Mean_Pos** | **Average_Mean_Neg** | **P-value** |
| --- | --- | --- | --- | --- |
| 1 | ExtFP151 | 0.054719562 | 0.169630643 | 0.00 |
| 2 | ExtFP153 | 0 | 0.006839945 | 0.68 |
| 3 | ExtFP191 | 0.653898769 | 0.902872777 | 0.00 |
| 4 | ExtFP351 | 0.838577291 | 0.511627907 | 0.00 |
| 5 | ExtFP369 | 0.846785226 | 0.55129959 | 0.00 |
| 6 | ExtFP841 | 0.701778386 | 0.928864569 | 0.00 |
| 7 | ExtFP956 | 0.009575923 | 0.108071135 | 0.00 |
| 8 | GraphFP624 | 0.009575923 | 0.165526676 | 0.00 |
| 9 | PubchemFP36 | 0.01504788 | 0.248974008 | 0.00 |
| 10 | SubFP1 | 0.719562244 | 0.94254446 | 0.00 |
| 11 | SubFP99 | 0.496580027 | 0.859097127 | 0.00 |
| 12 | SubFP100 | 0.980848153 | 0.848153215 | 0.00 |
| 13 | KRFP362 | 0 | 0.009575923 | 0.22 |
| 14 | KRFP841 | 0.132694938 | 0.461012312 | 0.00 |
| 15 | KRFP890 | 0.021887825 | 0.0875513 | 0.00 |
| 16 | KRFP1405 | 0.496580027 | 0.859097127 | 0.00 |
| 17 | SubFPC36 | 0.374829001 | 1.180574555 | 0.00 |
| 18 | SubFPC143 | 3.699042408 | 1.154582763 | 0.00 |
| 19 | SubFPC274 | 11.65253078 | 12.46511628 | 1.00 |
| 20 | SubFPC302 | 89.86320109 | 70.25854993 | 0.00 |
| 21 | KRFPC341 | 14.69357045 | 10.12995896 | 0.00 |
| 22 | KRFPC841 | 0.21751026 | 0.781121751 | 0.00 |
| 23 | KRFPC1146 | 4.031463748 | 2.123119015 | 0.00 |
| 24 | KRFPC1149 | 0.374829001 | 1.180574555 | 0.00 |
| 25 | KRFPC3013 | 3.410396717 | 0.801641587 | 0.00 |
| 26 | KRFPC3393 | 1.934336525 | 2.248974008 | 0.34 |
| 27 | KRFPC3706 | 14.96853625 | 12.27770178 | 0.00 |

**Table S4. Performance of different machine learning methods on amino acid composition.**

|  | **Parameters** | **Main Dataset** | | | | | **Validation Dataset** | | | | |
| --- | --- | --- | --- | --- | --- | --- | --- | --- | --- | --- | --- |
|  |  | **Sen** | **Spc** | **Acc** | **MCC** | **AUROC** | **Sen** | **Spc** | **Acc** | **MCC** | **AUROC** |
| SVM | g=0.001, c=8, j=2 | 93.81 | 89.52 | 91.67 | 0.83 | 0.96 | 92.00 | 87.33 | 89.67 | 0.79 | 0.96 |
| Random Forest | Ntree=170 | 95.36 | 89.18 | 92.27 | 0.85 | 0.97 | 95.33 | 80.67 | 88.00 | 0.77 | 0.95 |
| SMO | g=0.001, c=5 | 93.13 | 91.24 | 92.18 | 0.84 | 0.92 | 92.67 | 88.00 | 90.33 | 0.81 | 0.90 |
| J48 | c=0.3, m=1 | 88.83 | 86.94 | 87.89 | 0.76 | 0.87 | 90.00 | 88.00 | 89.00 | 0.78 | 0.88 |
| Naive Bayes | Default | 69.59 | 70.79 | 70.19 | 0.40 | 0.79 | 76.67 | 62.67 | 69.67 | 0.40 | 0.79 |

**Table S5.** **Performance of SVM method on amino acid composition features of terminus residues.**

|  | **Parameters** | | | **Main Dataset** | | | | | **Validation Dataset** | | | | |
| --- | --- | --- | --- | --- | --- | --- | --- | --- | --- | --- | --- | --- | --- |
|  | **G** | **c** | **j** | **Sen** | **Spc** | **Acc** | **MCC** | **AUROC** | **Sen** | **Spc** | **Acc** | **MCC** | **AUROC** |
| N5 | 0.0005 | 6 | 3 | 90.85 | 87.73 | 89.32 | 0.79 | 0.93 | 88.19 | 84.14 | 86.16 | 0.72 | 0.92 |
| N10 | 0.001 | 5 | 2 | 90.44 | 88.39 | 89.40 | 0.79 | 0.95 | 88.98 | 90.70 | 89.84 | 0.80 | 0.97 |
| N15 | 0.005 | 3 | 1 | 92.84 | 88.82 | 90.86 | 0.82 | 0.97 | 91.30 | 85.90 | 88.82 | 0.77 | 0.96 |
| C5 | 0.001 | 2 | 1 | 86.50 | 86.47 | 86.49 | 0.73 | 0.93 | 82.98 | 87.76 | 85.42 | 0.71 | 0.92 |
| C10 | 0.001 | 6 | 1 | 91.42 | 89.27 | 90.33 | 0.81 | 0.95 | 86.05 | 93.08 | 89.58 | 0.79 | 0.95 |
| C15 | 0.001 | 15 | 1 | 91.43 | 91.25 | 91.34 | 0.83 | 0.96 | 88.04 | 82.05 | 85.29 | 0.70 | 0.93 |
| N5C5 | 0.0005 | 1 | 4 | 89.29 | 87.78 | 88.45 | 0.77 | 0.94 | 89.52 | 88.03 | 88.72 | 0.77 | 0.95 |
| N10C10 | 0.001 | 2 | 1 | 92.95 | 91.90 | 92.37 | 0.85 | 0.97 | 95.00 | 89.15 | 91.97 | 0.84 | 0.96 |
| N15C15 | 0.001 | 4 | 1 | 92.53 | 90.39 | 91.37 | 0.83 | 0.96 | 90.80 | 84.62 | 87.88 | 0.76 | 0.95 |

**Table S6. Performance of different machine learning methods on dipeptide composition.**

|  | **Parameters** | **Main Dataset** | | | | | **Validation Dataset** | | | | |
| --- | --- | --- | --- | --- | --- | --- | --- | --- | --- | --- | --- |
|  |  | **Sen** | **Spc** | **Acc** | **MCC** | **AUROC** | **Sen** | **Spc** | **Acc** | **MCC** | **AUROC** |
| SVM | g=0.001, c=15, j=2 | 91.07 | 92.61 | 91.84 | 0.84 | 0.96 | 92.00 | 92.67 | 92.33 | 0.85 | 0.97 |
| Random Forest | Ntree=180 | 94.85 | 87.46 | 91.15 | 0.83 | 0.97 | 94.67 | 84.00 | 89.33 | 0.79 | 0.95 |
| SMO | g=0.0005, c=5 | 91.58 | 92.10 | 91.84 | 0.84 | 0.91 | 93.33 | 93.33 | 93.33 | 0.87 | 0.93 |
| J48 | c=0.2, m=5 | 85.40 | 84.36 | 84.88 | 0.70 | 0.89 | 86.00 | 86.00 | 86.00 | 0.72 | 0.90 |
| Naive Bayes | Default | 78.18 | 82.30 | 80.24 | 0.61 | 0.80 | 80.67 | 80.00 | 80.33 | 0.61 | 0.80 |

**Table S7. Performance of SVM method on dipeptide composition features of terminus residues.**

|  | **Parameters** | | | **Main Dataset** | | | | | **Validation Dataset** | | | | |
| --- | --- | --- | --- | --- | --- | --- | --- | --- | --- | --- | --- | --- | --- |
|  | **G** | **c** | **j** | **Sen** | **Spc** | **Acc** | **MCC** | **AUROC** | **Sen** | **Spc** | **Acc** | **MCC** | **AUROC** |
| N5 | 0.0005 | 1 | 3 | 90.67 | 87.73 | 89.23 | 0.78 | 0.96 | 89.58 | 86.90 | 88.24 | 0.77 | 0.95 |
| N10 | 0.001 | 3 | 1 | 92.72 | 87.78 | 90.23 | 0.81 | 0.97 | 96.06 | 90.70 | 93.36 | 0.87 | 0.98 |
| N15 | 0.001 | 3 | 3 | 92.84 | 89.71 | 91.29 | 0.83 | 0.97 | 92.39 | 88.46 | 90.59 | 0.81 | 0.95 |
| C5 | 0.0005 | 1 | 2 | 87.07 | 86.84 | 86.95 | 0.74 | 0.94 | 87.94 | 91.16 | 89.58 | 0.79 | 0.95 |
| C10 | 0.001 | 2 | 1 | 92.68 | 90.08 | 91.36 | 0.83 | 0.97 | 89.92 | 93.85 | 91.89 | 0.84 | 0.96 |
| C15 | 0.001 | 3 | 1 | 92.00 | 91.55 | 91.77 | 0.84 | 0.97 | 89.13 | 89.74 | 89.41 | 0.79 | 0.95 |
| N5C5 | 0.0005 | 1 | 1 | 90.82 | 90.22 | 90.49 | 0.81 | 0.96 | 91.13 | 92.25 | 91.73 | 0.83 | 0.96 |
| N10C10 | 0.0005 | 4 | 2 | 92.17 | 90.62 | 91.31 | 0.83 | 0.97 | 94.17 | 93.80 | 93.98 | 0.88 | 0.97 |
| N15C15 | 0.001 | 2 | 2 | 93.95 | 91.89 | 92.83 | 0.86 | 0.97 | 91.95 | 85.90 | 89.09 | 0.78 | 0.95 |
